# Generation Dependent Effects and Entrance to Mitochondria of Hybrid Dendrimers on Normal and Cancer Neuronal Cells In Vitro

**DOI:** 10.3390/biom10030427

**Published:** 2020-03-09

**Authors:** Aleksandra Szwed, Katarzyna Miłowska, Sylwia Michlewska, Silvia Moreno, Dzmitry Shcharbin, Rafael Gomez-Ramirez, Francisco Javier de la Mata, Jean-Pierre Majoral, Maria Bryszewska, Teresa Gabryelak

**Affiliations:** 1 Department of General Biophysics, Faculty of Biology and Environmental Protection, University of Lodz, 141/143 Pomorska Street, 90-236 Lodz, Poland; aleksandra.szwed@biol.uni.lodz.pl (A.S.); katarzyna.milowska@biol.uni.lodz.pl (K.M.); sylwia.michlewska@biol.uni.lodz.pl (S.M.); maria.bryszewska@biol.uni.lodz.pl (M.B.); teresa.gabryelak@biol.uni.lodz.pl (T.G.); 2 Departamento de Química Orgánica y Química Inorgánica, Universidad de Alcalá, Campus Universitario, E-28871 Alcalá de Henares, Spain; silvia_csld@hotmail.com (S.M.); rafael.gomez@uah.es (R.G.-R.); javier.delamata@uah.es (F.J.d.l.M.); 3 Networking Research Center on Bioengineering, Biomaterials and Nanomedicine (CIBER-BBN), E-28871 Alcalá de Henares, Spain; 4 Institute of Biophysics and Cell Engineering of NASB, Akademicheskaja, 27, 220072 Minsk, Belarus; 5 Laboratoire de Chimie de Coordinations du CNRS, 205 route de Narbonne, BP 44099 Toulouse CEDEX 4, France; majoral@lcc-toulouse.fr; 6 Department of Chemistry, Université de Toulouse, UPS, INPT, 31077 Toulouse CEDEX, France

**Keywords:** apoptosis, cytotoxicity, N2a, mHippoE-18, ROS, ΔΨm, TEM, carbosilane–viologen–phosphorus dendrimers

## Abstract

Dendrimers as drug carriers can be utilized for drugs and siRNA delivery in central nervous system (CNS) disorders, including various types of cancers, such as neuroblastomas and gliomas. They have also been considered as drugs per se, for example as anti-Alzheimer’s disease (AD), anti-cancer, anti-prion or anti-inflammatory agents. Since the influence of carbosilane–viologen–phosphorus dendrimers (SMT1 and SMT2) on the basic cellular processes of nerve cells had not been investigated, we examined the impact of two generations of these hybrid macromolecules on two murine cell lines—cancer cell line N2a (mouse neuroblastoma) and normal immortalized cell line mHippoE-18 (embryonic mouse hippocampal cell line). We examined alterations in cellular responses including the activity of mitochondrial dehydrogenases, the generation of reactive oxygen species (ROS), changes in mitochondrial membrane potential, and morphological modifications and fractions of apoptotic and dead cells. Our results show that both dendrimers at low concentrations affected the cancer cell line more than the normal one. Also, generation-dependent effects were found: the highest generation induced greater cytotoxic effects and morphological modifications. The most promising is that the changes in mitochondrial membrane potential and transmission electron microscopy (TEM) images indicate that dendrimer SMT1 can reach mitochondria. Thus, SMT1 and SMT2 seem to have potential as nanocarriers to mitochondria or anti-cancer drugs per se in CNS disorders.

## 1. Introduction

As the world’s population grows almost linearly, by 2050, the fraction of the population above the age of 65 years is expected to be approximately 16% [1]. In the ageing population, the incidence of neurological disorders such as Alzheimer’s disease (AD), Parkinson’s disease (PD), multiple sclerosis (MS), and primary brain tumors have a tendency to increase [2]. As it has been pointed out by Mignani et al., around one billion people suffer from disorders related to the central nervous system (CNS) [3]. Due to the immediate need to improve the therapy and diagnosis of psychiatric, developmental, traumatic, inflammatory, infectious and degenerative nervous system disorders, the interest in nanoneuromedicine is growing rapidly [2,4]. The main issue in the treatment and diagnosis of CNS disorders is to overcome the blood–brain barrier (BBB) without affecting its permeability. Impaired permeability of BBB promotes neuroinflammation and neurodegeneration and occurs in several high incidence pathologies such as stroke, AD and PD. The human BBB is composed of specialized tight junctions between endothelial cells that line brain capillaries. The most important feature of this dynamic barrier is to control brain homeostasis. Hence, the BBB protects our most delicate organ against the entry of intrusive chemicals as well as the delivery of drugs from the blood circulation [3,5,6]. Researchers have developed various ways to overcome the BBB. Physical methods typically use invasive neurosurgical approaches and an intracranial drug delivery system (DDS). These include techniques such as administering therapeutic agents directly into the cerebral ventricles of the brain, into the cerebrum, or into the cerebrospinal fluid, as well as intracerebral drug implants and convection-enhanced diffusion (into a specific cavity in the brain), or the use of catheters and other devices. Besides being highly toxic, the physical methods mentioned above have other disadvantages and limitations. Thus, an ideal strategy to overcome the BBB is one that is minimally invasive, clinically effective, and can be administered systemically in a patient-compliant and therapeutically effective manner [1,3,6]. One of the most promising drug delivery systems (DDS) into organs like the brain are nanoparticles (NPs). The most promising feature of NPs is that they are able to efficiently deliver therapeutic agents into difficult to reach regions and to provide the necessary protection to transported drugs. This is why nanoparticles are considered as one of the most auspicious and versatile drug delivery systems [7]. Dendrimers, which are the subject of these studies, distinguish themselves from other nanoparticles due to their monodispersity, high biocompatibility and good water solubility. These highly branched and tunable globular macromolecules have been well described both for therapeutic applications and diagnostics [3,8,9]. As highlighted by Mignani et al. [3,10], dendrimers can be administered by many different routes, including: intravenous, intraperitoneal, ocular, transdermal, oral, intranasal, pulmonary, intravaginal and transmucosal [3,10]. Dendrimers as drug carriers can be utilized for drugs and siRNA delivery in CNS disorders, including various types of cancers, such as neuroblastomas and gliomas. It has been shown that conjugation of these nanostructures with D-glucosamine results in increasing endocytosis and permeability across the BBB and tumor targeting [11,12]. Moreover, dendrimers can also be considered as drugs per se; for example as anti-AD, anti-cancer, anti-prion or anti-inflammatory agents [3].

In this paper, two generations of hybrid carbosilane–viologen–phosphorus dendrimers were examined. The synthesis of these hybrid dendrimers, called SMT1 and SMT2, was based on the “onion peel” approach. Carbosilane–viologen–phosphorus dendrimers have two kinds of cationic groups: those located at the branches due to viologen quaternized units and those related to the ammonium groups at the surface of carbosilane wedges [9].

The aim of this study was to investigate whether carbosilane–viologen–phosphorus dendrimers of two generations have potential as nanocarriers or drugs per se in CNS disorders. To understand events occurring in two different murine cell lines, N2a (mouse neuroblastoma) and mHippoE-18 (embryonic mouse hippocampal cell line), after 24 h of SMT treatment, we examined alterations in cellular responses including activity of mitochondrial dehydrogenases, generation of reactive oxygen species (ROS), changes in mitochondrial membrane potential, morphological modifications and fractions of apoptotic and dead cells.

## 2. Materials and Methods

### 2.1. Dendrimers

**Figure 1:**
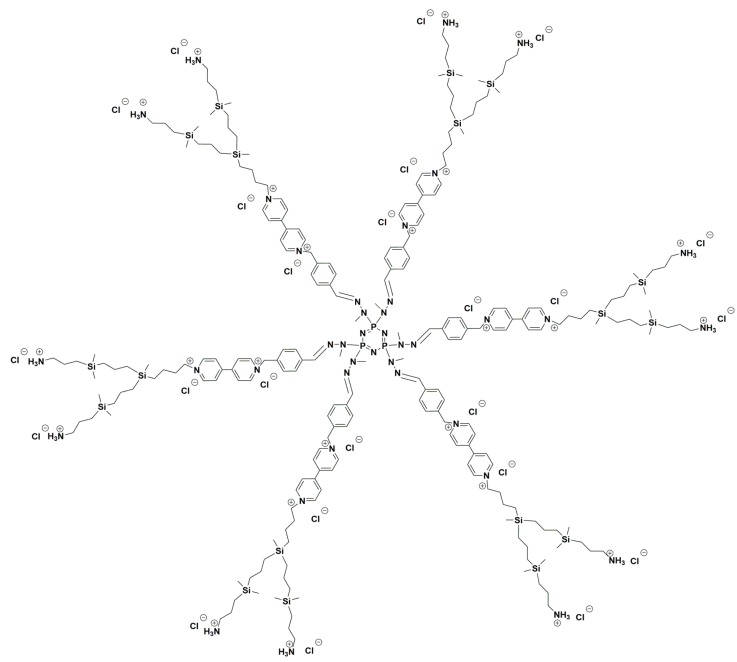

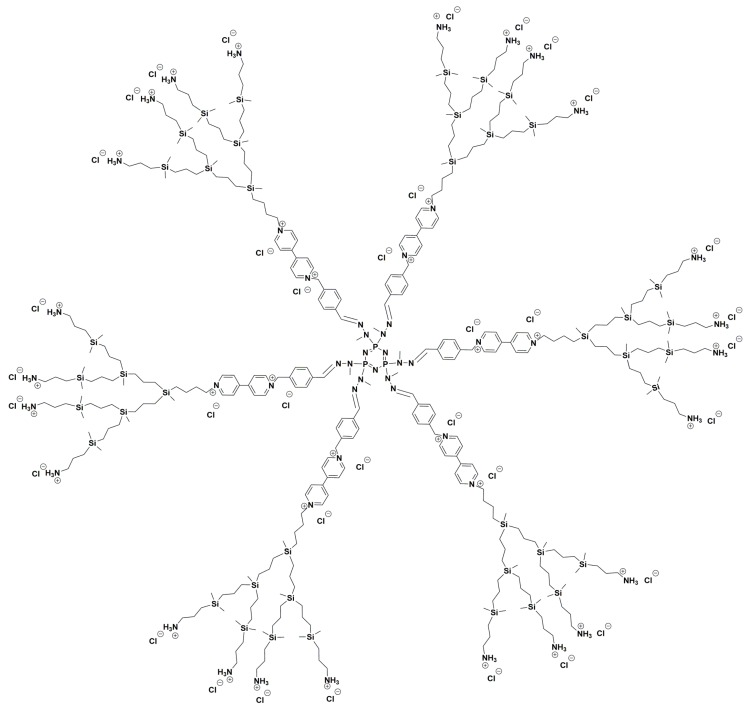
Chemical structures of dendrimers SMT1 and SMT2 [9].

Cationic dendrimers SMT1 and SMT2 were synthesized in Departamento de Química Orgánica y Química Inorgánica, Universidad de Alcalá (Spain) [9] (Figure 1).

SMT1: {N3P3[(Viologen)G1CBS(NH3)2]6}(Cl)24 **[9]**.

SMT2: {N3P3[(Viologen)G2CBS(NH3)4]6}(Cl)36 [9].

### 2.2. Cell Culture

The mHippoE-18 cell line (embryonic mouse hippocampal cell line) was purchased from Cederlane (Burlington, Ontario, Canada), whereas the N2a cell line (mouse neuroblastoma cell line) was purchased from the American Type Culture Collection (ATCC, VA, USA) (CCL-131). Cells were cultured in DMEM medium supplemented with 5% fetal bovine serum and incubated at 37 °C in an atmosphere of 5% CO_2_. They were split for subcultures every 2–3 days.

### 2.3. Cytotoxicity Evaluation

The cytotoxicity of carbosilane–viologen–phosphorus dendrimers was evaluated using the 3-(4,5-dimethylthiazol-2-yl)-2,5-diphenyltetrazolium bromide (MTT) assay. The assay is based on the reduction of MTT by cellular reductases of viable cells to a blue formazan product, whose absorbance can be measured spectrophotometrically after solubilization [13]. Cells (1.5 × 10^4^ cells/well) were seeded in 96-well transparent plates with 100 μL of DMEM medium and they were cultured for 20 h under growing conditions for cell attachment, then treated with dendrimers and incubated for a further 24 h under growing conditions. After incubation with dendrimers, 0.5 mg/mL MTT was added to each well and incubated for 3 h at 37 °C. After this time, the MTT solution was discarded, dimethyl sulfoxide (DMSO) was added to each well to dissolve the formazan crystals, and the absorbance was measured at 570 nm using a microplate reader BIOTEK PowerWave HT (BioTek, VT, USA).

### 2.4. Measurement of Reactive Oxygen Species (ROS)

Changes in the intracellular level of reactive oxygen species (ROS) were investigated by the fluorescent probe 2′,7′-dichlorodihydrofluorescein diacetate (H_2_DCFDA; Molecular Probes™, ThermoFisher Scientific, USA). H_2_DCFDA does not emit fluorescence until it enters the cell, where the acetate groups are removed by intracellular esterases. Subsequently, upon oxidation, non-fluorescent H_2_DCF is converted to a highly fluorescent form—dichlorofluorescein (DCF) [14]. The amount of the intracellular ROS is proportional to the intensity of DCF.

In this study, cells (1.5 × 10^4^ cells/well) were seeded in 96-well black plates with 100 μL of DMEM medium. They were cultured for 20 h under growing conditions for cell attachment, then treated with dendrimers and incubated for a further 24 h under growing conditions. After incubation with dendrimers, the cells were stained with 50 μL of 2 μM H_2_DCFDA for 15 min under growing conditions. After the probe solution was discarded, the cells were washed with PBS and 100 μL of PBS was added. The fluorescence of DCF was measured at 485/530 nm using a microplate reader BIOTEK PowerWave HT (BioTek, VT, USA).

### 2.5. Assessment of Mitochondrial Membrane Potential (Ψm)

To determine mitochondrial membrane potential (Ψm), the fluorescent dye 5,5′,6,6′-tetrachloro-1,1′,3,3′-tetraethylbenzimidazolylcarbocyanine iodide (JC-1) was used. JC-1 is a lipophilic cationic dye which accumulates in mitochondria. At higher concentrations, JC-1 forms so called J-aggregates, which exhibit red fluorescence (λex = 530 nm, λem = 590 nm). In the case of mitochondrial membrane depolarization, the dye does not form J-aggregates and exists in the form of monomers, which exhibit green fluorescence (λex = 485 nm, λem = 538 nm). The loss of Ψm can be indicated by a decrease in the red to green fluorescence intensity ratio [15]. Cells (1.5 × 10^4^ cells/well) were seeded in 96-well black plates with 100 μL of DMEM medium. They were cultured for 20 h under growing conditions for cell attachment, then treated with dendrimers and incubated for a further 24 h under growing conditions. After incubation with dendrimers, the cells were stained with 50 μL of 5 μM JC-1 in each well and incubated for 20 min under growing conditions. The dye was discarded, cells were washed with PBS and 100 μL of PBS was added to each well. The fluorescence was measured using a microplate reader BIOTEK PowerWave HT (BioTek, VT, USA).

### 2.6. Visualisation of Cell Morphology by Confocal Microscopy Imaging

The morphology of the mHippoE-18 and N2a cells after 24-h treatment with the SMT1 dendrimer at the concentration range of 0.1–10 μM was assessed using confocal microscopy. Cells (2.5 × 10^5^ cells/mL) were seeded and grown in DMEM with 5% heat inactivated FBS at 37 °C in a humidified atmosphere containing 5% CO_2_/95% air. After incubation, the cells were gently washed twice with 0.1 M phosphate-buffered saline (PBS; pH 7.2) and fixed in 3% formaldehyde for 2 h. Subsequently, the cells were washed with PBS and stained with DAPI and Texas Red-X Phalloidin. Images were taken with a Leica TCS SP8 microscope at two wavelengths (405 nm and 565 nm). Leica software (Wetzlar, Germany) was used to analyze the data.

### 2.7. Determination of Apoptosis and Necrosis by OA/EB Double Staining—Confocal Microscopy Imaging

Orange acridine (OA) and ethidium bromide (EB) double staining was carried out [16]. Both dyes exhibit fluorescence after intercalation in DNA. However, OA is taken up by all cells and stains the nucleus green, whereas EB enters only cells with damaged membranes and stains the nucleus red. Thus, it is possible to identify separate fractions of cells where viable cells are morphologically normal with a green nucleus, early apoptotic cells have a green nucleus with condensed or fragmented chromatin, late apoptotic cells have condensed or fragmented red chromatin, and necrotic cells are morphologically normal with a red nucleus. In this study, the dyes were added to each sample at a concentration of 2 µg/mL for 2 min. Afterwards, the cells were washed twice with PBS and visualized with a Leica TCS SP8 microscope (Wetzlar, Germany).

### 2.8. Determination of Apoptotic and Dead Cells by Annexin V Staining-Flow Cytometry Analysis

To determine apoptotic and dead cells, the annexin-V (Ann-V)/propidium iodide (PI) assay was performed. Annexin-V is a Ca^2+^-dependent phospholipid-binding protein with high affinity for phosphatidylserine (PS), which, in apoptotic cells, is translocated from the inner to the outer leaflet of the plasma membrane. Thereby, PS exposed to the external cellular environment can be bound by annexin-V. Propidium iodide is excluded by viable cells with intact membranes, whereas the membranes of dead and damaged cells are permeable to PI. Therefore, separate fractions of cells were identified as follows: viable cells—Ann-V and propidium iodide negative; necrotic cells—Ann-V negative and PI positive; late apoptotic cells—Ann-V and propidium iodide positive; early apoptotic cells—Ann-V positive and PI negative. Samples were prepared according to the manufacturer’s instructions for the FITC Annexin-V Apoptosis Detection Kit I (BD Pharmingen™). Data were recorded for a total of 10,000 events per sample. Compensation controls for annexin were treated with camptothecin (80 µM) for 4 h (positive green fluorescence), and for propidium iodide, the cells were treated with frozen 75% ethanol (positive red fluorescence). The samples were analyzed by flow cytometry (LSRII, Becton Dickinson, NJ, USA).

### 2.9. TEM

TEM analysis was conducted to evaluate changes in the N2a cell ultrastructure. Cells (2.5 × 10^5^ cells/mL) were grown in DMEM with 5% heat inactivated FBS at 37 °C in a humidified atmosphere containing 5% CO_2_/95% air. The cells were treated with 5μM SMT1 and SMT2 dendrimers and incubated for 24 h. After that time, the cells were gently washed with 0.1 M PBS (pH 7.2). Then, the cells were fixed with 2.5% glutaraldehyde in the same PBS buffer for 2 h. Next, they were scraped off and dispersed into 1.5% agarose and rinsed with buffer three times. The agar blocks were postfixed in 1% osmium tetroxide for 2 h at 4 °C. After this, the material was dehydrated in a graded series of ethanol, then propylene oxide. Subsequently, the cells were embedded in Epon-Spur’s resin mixture. Sample resin blocks were sectioned on an Ultra Cut E (Reichert Jung, Germany) ultramicrotome with a glass knife. Ultrathin sections (60–70 nm) were placed on formvar coated nickel grids and stained with a uranyl acetate and lead citrate [16,17]. The cell ultrastructure was examined using a transmission electron microscope JEM 1010 (JEOL, Japan) at 80 kV.

### 2.10. Statistical Analysis

The results were subjected to statistical analysis with the use of the t-test (in the case of normal distribution) or the Mann–Whitney Rank Sum Test (in the case of lack of normal distribution). To assess the significance of differences between particular dendrimers, the ANOVA test was conducted. In the case of differences, further analysis was performed using the Luke’s method. In all cases, the level of significance α = 0.05 or less.

## 3. Results

### 3.1. Cytotoxicity Evaluation

**Figure 2:**
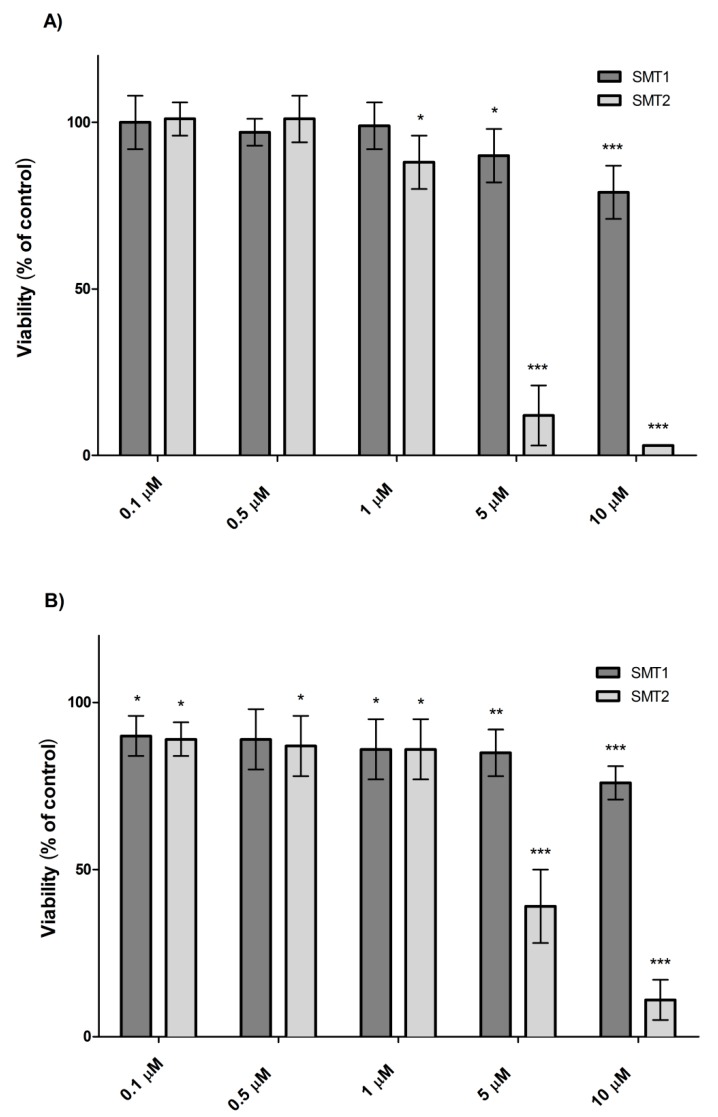
Viability of the mHippoE-18 (**A**) and N2a (**B**) cells after 24 h exposure to SMT, determined using the 3-(4,5-dimethylthiazol-2-yl)-2,5-diphenyltetrazolium bromide (MTT) test (*n* = 6, * *p* < 0.05, ** *p* < 0.01, *** *p* < 0.001 in relation to the control).

**Figure 3:**
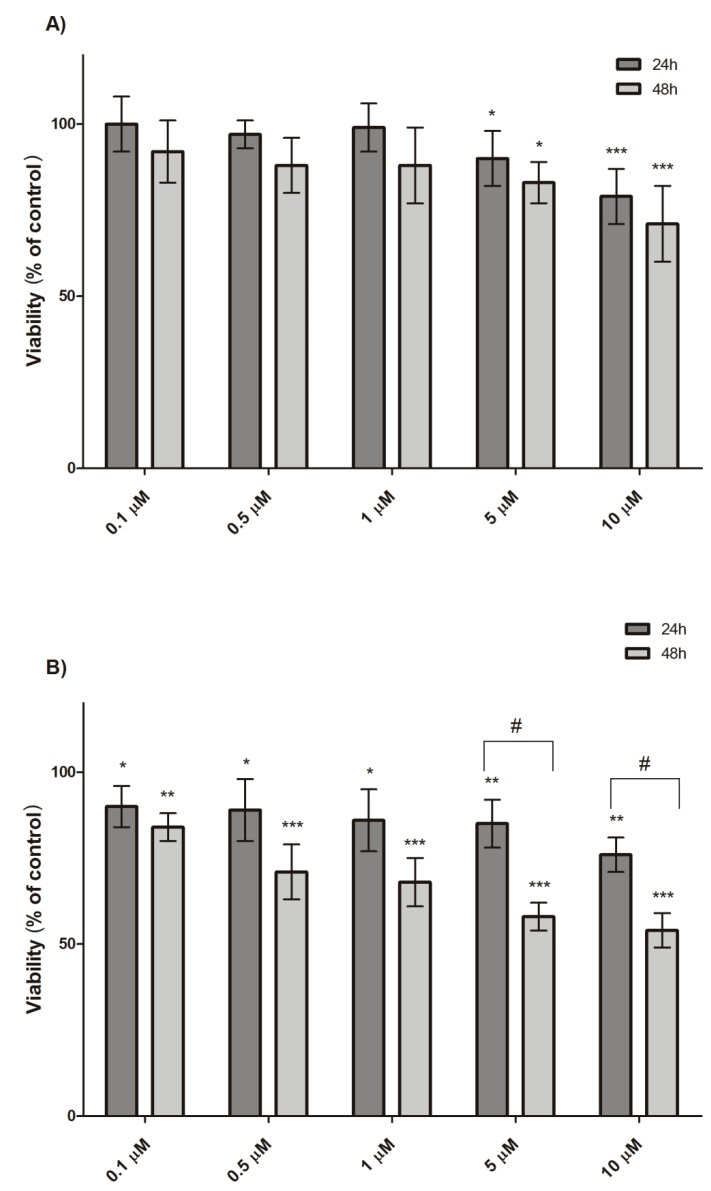
Viability of the mHippoE-18 (**A**) and N2a (**B**) cells after 24 h and 48 h exposure to SMT1, determined using the MTT test (*n* = 6, * *p* < 0.05, ** *p* < 0.01, *** *p* < 0.001, # *p* < 0.001 in relation to the control).

The standard MTT cytotoxicity assay revealed that the viability of mHippoE-18 and N2a cells, after 24 h incubation with dendrimer SMT1 only slightly decreased, reaching the level of 79% (mHippoE-18) and 76% (N2a) of the control at the highest concentration used (10 μM) (Figure 2 and Figure 3). Dendrimer SMT2, after 24 h incubation, caused a concentration-dependent decrease in cell viability. The 50% cytotoxic concentration (CC50) was defined as the dendrimer concentration that reduced the cell viability by 50% in relation to the untreated control. A CC50 value was calculated only for SMT2 dendrimers, due to minor toxicity of the first generation dendrimer. The CC50 value for SMT2 and mHippoE-18 cells was 2.32 ± 1.15 μM, whereas for N2a cells, it was 3.03 ± 1.08 μM. The results indicate that cells after 24 h treatment are more sensitive to exposure to the second generation of carbosilane–viologen–phosphorus dendrimers.

Due to the minor cytotoxicity of the SMT1 dendrimer after 24 h, the incubation time was extended to 48 h. The obtained results show that, in the case of N2a cells, the cytotoxicity of the SMT1 dendrimer increased and was concentration-dependent. There was a statistically significant difference in the viability of N2a cells between the treatment times at 24 h and 48 h for the concentrations 5 μM (up to 58%) and 10 μM (up to 54%) (Figure 3). The CC50 value of SMT1 dendrimers for N2a cells after 48 h of incubation was 14.34 ± 1.82 μM. In the case of mHippoE-18 cells, a slight decrease in viability after 48 h was observed; however, the decrease was not statistically significant compared to the results obtained after 24 h incubation.

### 3.2. Measurement of Reactive Oxygen Species (ROS)

**Figure 4:**
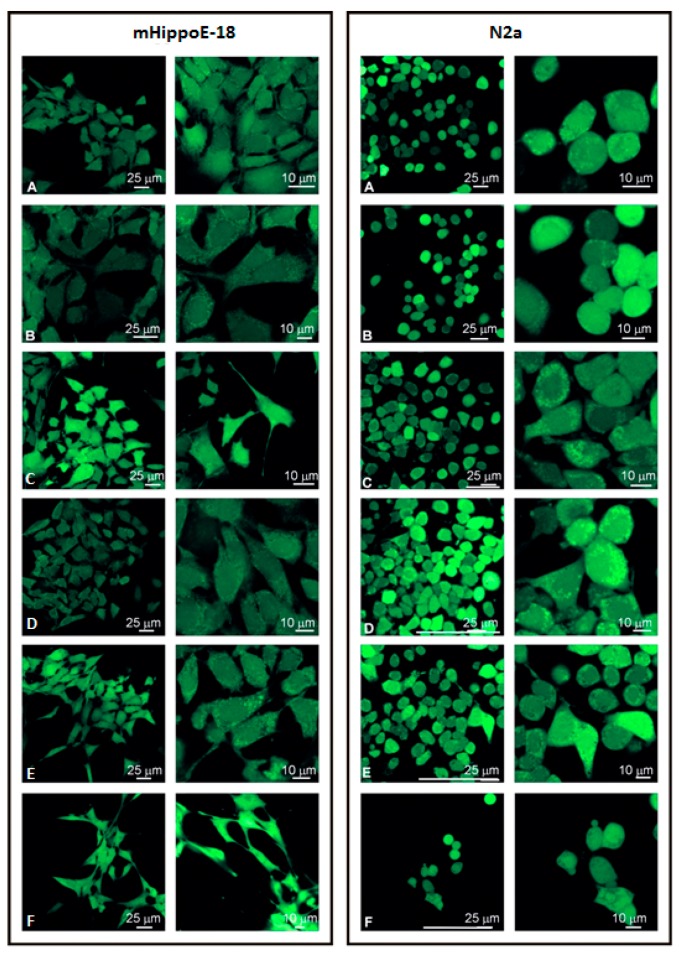
Changes in the level of reactive oxygen species (ROS) in mHippoE-18 and N2a cells after 24 h exposure to SMT1 visualized by confocal microscopy using the 2′,7′-dichlorodihydrofluorescein diacetate (H_2_DCFDA) probe. (**A**) Control; (**B**) 0.1 μM; (**C**) 0.5 μM; (**D**) 1 μM; (**E**) 5 μM; (**F**) 10 μM.

Alterations in the reactive oxygen species were assessed using the fluorescent probe H_2_DCFDA. After 24 h of incubation, there were no significant changes in the level of ROS for both cell lines compared to the control (data not presented). The ROS level in the cells incubated with the highly cytotoxic SMT2 dendrimer was evaluated only up to the concentration of 5 μM. The samples after 24 h treatment with the SMT1 dendrimer were also visualized by confocal microscopy (Figure 4). Visualization of N2a and mHippoE-18 cells confirmed the results obtained using a microplate reader BIOTEK PowerWave HT (BioTek, USA).

### 3.3. Alteration in Mitochondrial Membrane Potential (Ψm)

**Figure 5:**
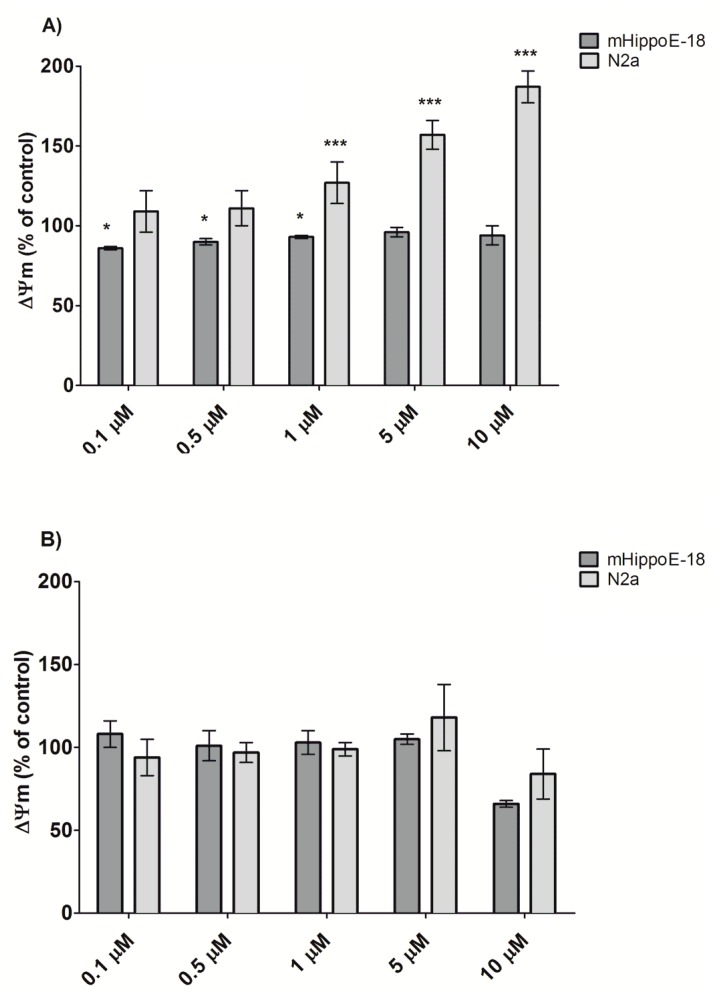
Alteration in the mitochondrial membrane potential (ΔΨm) in mHippoE-18 and N2a cells after 24 h exposure to SMT. (**A**) SMT1; (**B**) SMT2, determined using 5,5′,6,6′-tetrachloro-1,1′,3,3′-tetraethylbenzimidazolylcarbocyanine iodide (JC-1) fluorescent dye (*n* = 6, * *p* < 0.05, ** *p* < 0.01, *** *p* < 0.001 in relation to the control).

After the measurement of reactive oxygen species formation, changes in the mitochondrial membrane potential were evaluated using the JC-1 fluorescent probe (Figure 5). Due to the lack of significant changes in ROS production after 24 h incubation with the two SMT dendrimers, alterations in transmembrane mitochondrial potential were not expected. Surprisingly, SMT1 treatment for the N2a cell line caused perturbations in Ψm. After 24 h incubation, hyperpolarization of the mitochondrial membrane (up to 192% of the control at the highest concentration) was observed. For the mHippoE-18 cell line, Ψm slightly decreased in the lowest concentration of SMT1. In the case of SMT2, similarly to the ROS level measurement, ΔΨm was evaluated up to a concentration of 1 μM. There were no significant changes in the mitochondrial membrane potential for both cell lines after 24 h treatment (see Figure 5).

### 3.4. Visualisation of Cell Morphology by Confocal Microscopy Imaging

**Figure 6:**
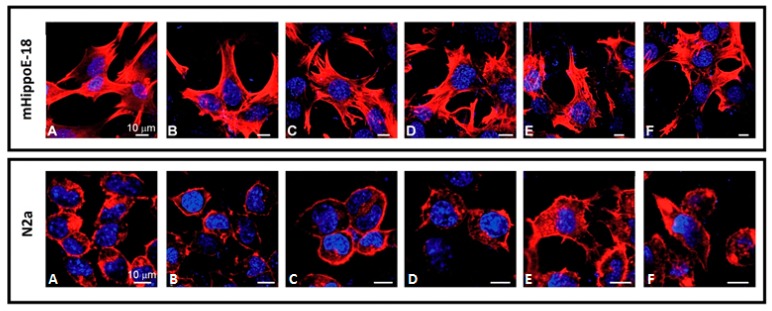
mHippoE-18 and N2a cell morphology after incubation with SMT1. The cells were stained with 4′,6-diamidino-2-phenylindole (DAPI) and Texas Red-X Phalloidin. Images were taken with a Leica TCS SP8 microscope at two wavelengths (405 nm and 565 nm). Bar = 10μm. (**A**) Control; (**B**) 0.1 μM; (**C**) 0.5 μM; (**D**) 1 μM; (**E**) 5 μM; (**F**) 10 μM.

Cell morphology was analyzed using a confocal microscope. The microscopic network of microtubules was visualized with phalloidin, while the cell nucleus was stained by DAPI (Figure 6). The morphology of mHippoE-18 cells did not change after 24 h incubation with SMT1 in the concentration range from 0.1 μM to 10 μM. In contrast, changes in the morphology of N2a cells were observed. After incubation with SMT1 at 5 μM and 10 μM, the cells were bigger. Moreover, disorganization of the cytoskeleton was observed and additional cellular extensions were revealed. Most likely, slight chromatin condensation also occurred.

### 3.5. Determination of Apoptosis and Necrosis by Orange Acridine/Ethidium Bromide (OA/EB) Double Staining—Confocal Microscopy Imaging

**Figure 7:**
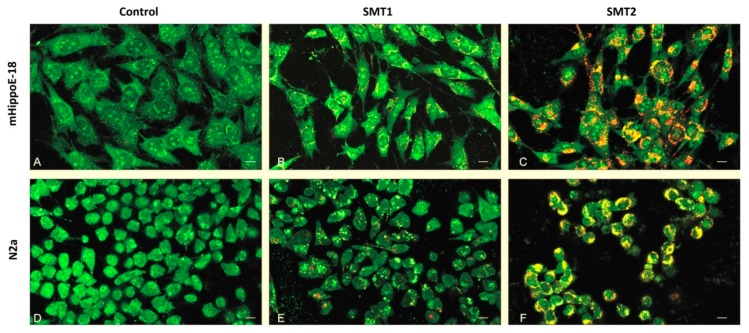
Images of mHippoE-18 (**A**–**C**) and N2a (**D**–**F**) cells after 24 h exposure to 5 μM SMT1 (**B** and **E**) and SMT2 (**C** and **F**). Cells were stained with orange acridine (OA) and ethidium bromide (EB) and examined by confocal microscopy. Viable cells: morphologically normal with green nucleus; early apoptotic cells: green nucleus with condensed or fragmented chromatin; late apoptotic cells: condensed or fragmented red chromatin; necrotic cells: morphologically normal with red nucleus.

The above described alterations in mitochondrial function, the viability of cells, and changes in their morphology after 24 h treatment with 5 μM SMT1 were connected with the induction of apoptosis in neuroblastoma cells. Confocal analysis of OA/EB stained samples confirmed high toxicity and revealed strong morphological changes after 24 h incubation with SMT2 for both cell lines (Figure 7). Data collected from confocal OA/EB stained cell images allowed for determining the fractions of healthy, necrotic, early and late apoptotic cells (Table 1). The obtained results showed that 5 μM of SMT1 caused a statistically significant increase (up to 15.95%) in the early apoptotic cell fraction of N2a cells compared to the control and mHippoE-18 cells. It led to a decrease in the healthy cell fraction of N2a (to 83.01%). In contrast, SMT2 dendrimers significantly increased the fraction of early apoptotic cells, simultaneously decreasing the fraction of healthy cells in N2a (up to 28.57%) and mHippoE-18 (up to 49.81%). The results correspond well with the cytotoxicity evaluation.

### 3.6. Determination of Apoptotic and Dead Cells by annexin-V and propidium iodide Staining—Flow Cytometry Analysis

**Figure 8:**
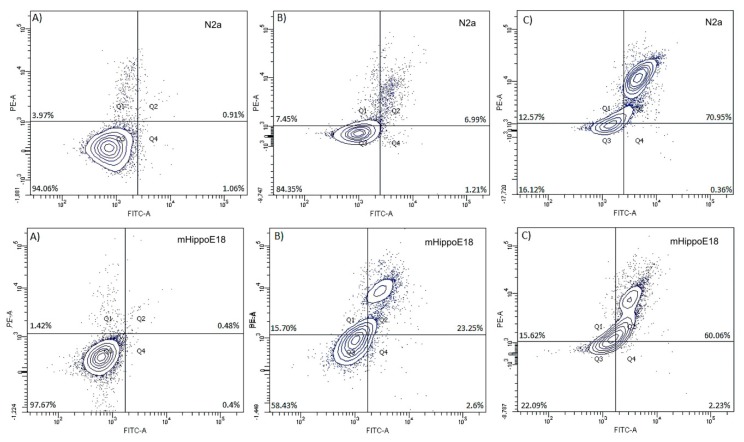
Representative dot plots showing fractions of necrotic (Q1), late apoptotic (Q2), healthy (Q3) and early apoptotic (Q4) of N2a and mHippoE-18 cells after 24 h exposure to 5 μM carbosilane–viologen–phosphorus dendrimers. Flow cytometry analysis was performed using annexin V and propidium iodide stains. (**A**) Control; (**B**) cells treated with SMT1; (**C**) cells treated with SMT2.

The results obtained previously have been confirmed by flow cytometry analysis of apoptotic and necrotic cells (Figure 8). Flow cytometry analysis of double-stained (Annexin V/PI) samples revealed that after 5 μM SMT treatment, the fraction of viable cells decreased from 97.67% for the control to 61.63% and 16.08% for the first and second generation, respectively. Simultaneously, the population of apoptotic cells at this concentration increased from 0.92% for the control to 23.3% (SMT1) and 69.32% (SMT2). SMT application to mHippoE-18 cells led to a slight fall (up to 77.16%) in the percentage of viable cells for SMT1 and a slight increase in the fraction of apoptotic cells (up to 10.36%). The effect of SMT2 on the hippocampal cells was similar to N2a cells. The fraction of viable and apoptotic cells changed drastically in comparison to the control. The amount of viable cells decreased to 18.92% and the apoptotic cell fraction increased up to 64.57% (Table 2). The results correspond well with the cytotoxicity evaluation. Slight differences may be caused by the disparity of the applied methods.

### 3.7. TEM—Ultra-Thin Sections

TEM analysis was conducted to assess ultrastructural changes in N2a cells under the influence of SMT1 and SMT2 dendrimers.

**Figure 9:**
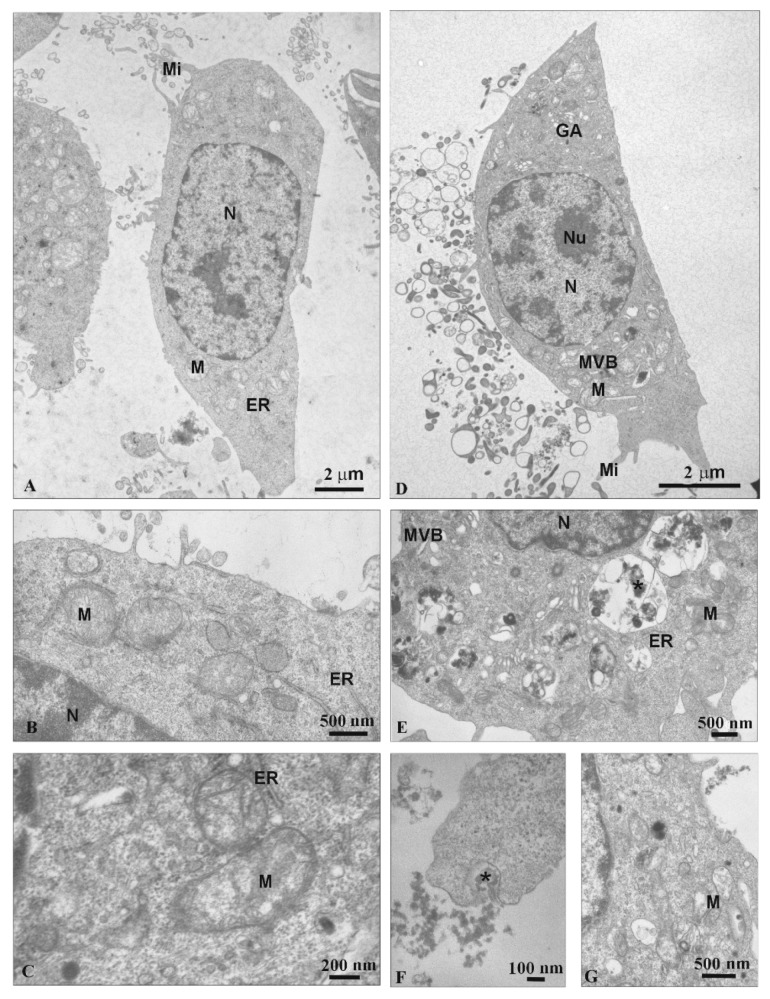
The ultrastructure of N2a cells. (**A**–**C**) Control; (**D**–**G**) 5 μM SMT1 dendrimer. N: nucleus; Nu: nucleolus; M: mitochondrion; MVB: multi-vesicular bodies; LB: lamellar bodies; GA: Golgi apparatus; ER: endoplasmic reticulum; Mi: microvilli; SL: secondary lysosomes; *: electron dense granular material.

Untreated cells had a normal ultrastructure, numerous microvilli (Mi), endoplasmic reticulum (ER) and mitochondria (M) in the cytoplasm (Figure 9A–C) and evenly distributed chromatin in the nucleus (N) were observed (Figure 9A).

The ultrastructure of cells treated with SMT1 was slightly changed (Figure 9D). Multi-vesicular bodies (MVB) and lamellar bodies (LB) appeared in the cells (Figure 9D,E). Moreover, under the influence of the SMT1 dendrimer, the secondary lysosomes (SL) were formed with electron dense granular material (*) (Figure 9E). Additionally, the mitochondrial matrix was sparse (Figure 9E,G).

**Figure 10:**
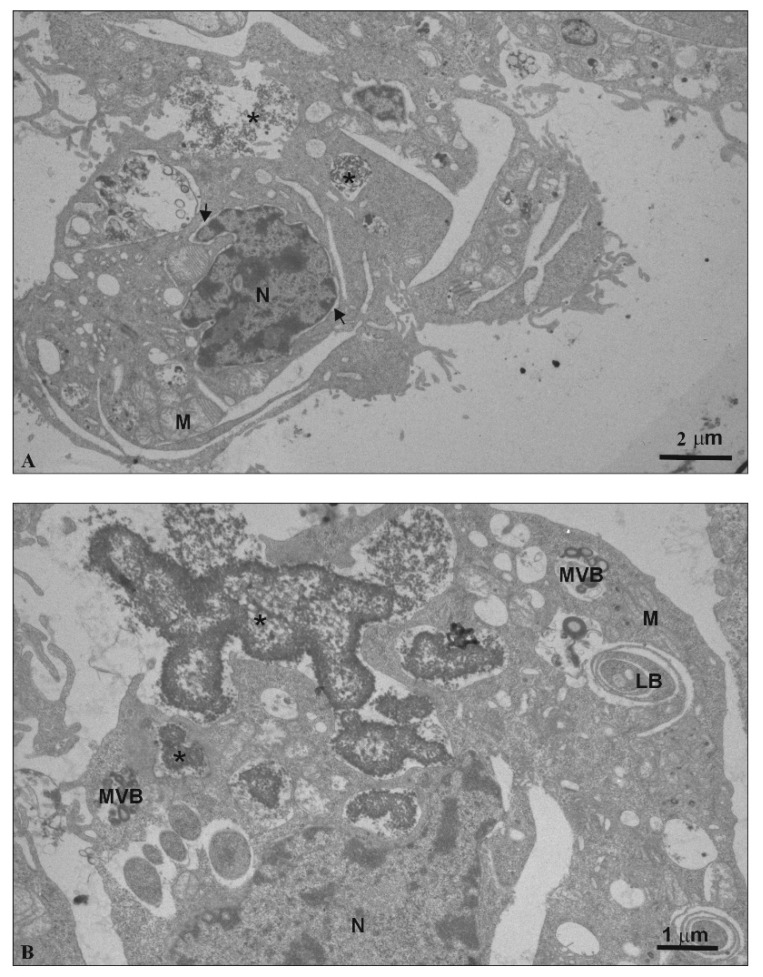
The ultrastructure of N2a cells under the influence of 5 μM SMT2 (**A** — bar 2 µm; **B** — bar 1 µm). N: nucleus; M: mitochondrion; MVB: multi-vesicular bodies; LB: lamellar bodies; ↑: separation of nuclear lamina from chromatin; *: electron dense granular material.

In contrast, the ultrastructure of cells treated with SMT2 was significantly changed (Figure 10A,B). Chromatin marginalization and condensation, the separation of nuclear lamina from chromatin, lamellar bodies, numerous secondary lysosomes and multi-vesicular bodies were observed in the cells (Figure 10A). Furthermore, the mitochondria were swollen, and they had disorganized mitochondrial cristae (Figure 10A).

Additionally, the endocytosis process was observed for cells treated with both dendrimers (Figure 9F and Figure 10B). While the SMT1 dendrimers were uptaken as single nanoparticles (*) (Figure 9F), SMT2 dendrimers created huge aggregated clusters (*) (Figure 10B).

## 4. Discussion

Nanostructures, including dendrimers, elicit complex biological responses and are immediately coated with proteins in biological fluids, forming a so-called “protein corona” [18]. Carbosilane–viologen–phosphorus dendrimers, the subject of this work, have already been studied in the context of their interaction with different types of proteins and their cytotoxicity to the Chinese hamster B14 cell line [9,18,19].

The goal of this study has been to investigate whether first and second generation carbosilane–viologen–phosphorus (SMT) dendrimers are potentially nanocarriers or anti-cancer drugs per se in CNS disorders. To understand events occurring in two different murine cell lines, N2a (mouse neuroblastoma) and mHippoE-18 (embryonic mouse hippocampal cell line), after 24 h of treatment with SMT1 or 2, the alterations in cellular responses, including the activity of mitochondrial dehydrogenases, generation of reactive oxygen species (ROS), changes in mitochondrial membrane potential, morphological modifications, and fractions of apoptotic and dead cells, were measured.

Cells were cultured in the presence of fetal bovine serum (FBS). Zarros et al. [20] suggested that experiments on the mHippoE-18 cell line grown without FBS is a more reliable way to assess neurotoxicity, and therefore we decided to reduce the FBS amount from 10% to 5%. Indeed, FBS affects dendrimer bioavailability and stimulates cell proliferation. Therefore, consistent conditions [20,21] were explored by decreasing the FBS amount since both cell lines, mHippoE-18 (which should represent a non-proliferating neuronal population) and cancer neuroblastoma (N2a) were being used. Navya and Daima [22] also pointed out that designer-made nanomaterials, according to their dynamic physicochemical and surface properties, can produce a series of interactions with biological entities [22]. Thus, the processes occurring in vivo seem to us to be so complex and multivariate that there would be no need to simplify them in in vitro studies by total FBS deprivation. Janaszewska et al. [21] had already shown that total deprivation of FBS in culture media prior to dendrimer treatment had no significant effect.

Our first step was to measure the cytotoxicity of SMT1 and SMT2 dendrimers in selected murine cell lines; the MTT assay after SMT treatment had already been run on the B14 (Chinese hamster) cell line [9]. The first generation of dendrimers, SMT1, was less toxic than SMT2 because the CC50 value for B14 cells at 24 h was >10 μM, whereas for SMT2 it was ~1 μM [9]. SMT2 caused a significant concentration-dependent loss of cell viability in N2a (CC50 ≈ 3.03 μM) and mHippoE-18 (CC50 ≈ 2.32 μM), whereas the SMT1 dendrimer at concentrations up to 10 μM was only slightly toxic for both lines after 24 h. However, after the incubation time was extended up to 48 h, the toxicity of SMT1 in N2a increased (CC50 ≈ 14.34μM) and a concentration-dependent loss of cell viability was observed. The difference between 24 h and 48 h incubation with 5 μM and 10 μM dendrimer for N2a cells was statistically significant. There was no such effect observed for the mHippoE-18 cell line. The data show that SMT1 and SMT2 dendrimers are more cytotoxic to N2a cancer cells than to normal cells.

Regarding the cytotoxicity of dendrimers, several reports indicate a strong correlation between toxicity and intracellular ROS production [23,24,25]. Increase in ROS is biphasic, with the first phase increase being attributed to the active uptake of the dendrimer (in this case polyamidoamine (PAMAM)) by clathrin-mediated endocytosis [25,26,27]. The second phase is due to mitochondrial injury as PAMAM localizes to these organelles [25,28]. Cytotoxicity has already been found to be correlated with increased ROS production; for example, for PAMAM and cationic phosphorus dendrimers (CPD) on N2a cells [29], whereas viologen–phosphorus dendrimers (VPD) only slightly decreased the ROS level in mHippoE-18 and N2a cells [30]. Carbosilane dendrimers had no statistically significant effect on the ROS level in mHippoE-18 [31]. SMT1 in the range of 0.1–10 μM and SMT2 in the range of 0.1–5 μM did not cause significant changes in ROS production for the mHippoE-18 and N2a cell lines. These results were confirmed by confocal microscopy. These results are in good correlation with data on apoptosis induced these cells.

Using the H2DCFDA probe, changes in mitochondrial potentials were not expected, which was confirmed for SMT2. However, after 24 h treatment with SMT1, a concentration-dependent increase in mitochondrial membrane potential occurred in N2a cells. Also, TEM images (Figure 9E,F) directly show the entrance of SMT1 into mitochondria. SMT1 and SMT2 at high concentrations induced some mitochondrial degradation (sparse mitochondrial matrix). VPD treatment for the mHipppoE-18 cell line perturbed Ψm, whereas mitochondrial activity remained unchanged or was slightly decreased in N2a cells [30]. Alterations—usually a collapse of Ψm—seem to be associated with mitochondria failure, leading to cell death. However, hyperpolarization of the mitochondrial membrane has also been described [32,33]. We have chosen the concentration of 5 μM for SMT1. This concentration had no significant effect on mHippoE-18 cells but affected N2a cells. At fixed concentration, changes in the transmembrane mitochondrial potential and the ROS level within the time range 0.5–48 h (data not shown) were recorded. N2a cells at 0.5 h showed a significant increase in ROS (up to ~120%) and ΔΨm (up to ~170%). At the 48 h end point, a significant collapse in both parameters to ~80% of the control value was observed. These results correspond with the cytotoxicity data, and loss of N2a cell viability up to 58%. For mHippoE-18 cells, no significant changes were observed over time. Based on this information, the first-generation dendrimer, SMT1, could be a good candidate as a drug per se or a drug carrier targeting mitochondria.

Analysis of mHippoE-18 cell morphology after 24 h treatment with SMT1 showed no changes, whereas at 5 μM and 10 μM, cellular extensions were seen, and most likely some slight changes in chromatin concentration occurred. Similarly, analysis of N2a cell ultrastructure after 24 h treatment with 5 μM hybrid dendrimers showed that SMT1 caused only slight changes, whereas SMT2 produced significant chromatin marginalization and condensation, as well as separation of nuclear lamina from chromatin. The same effect had been noted earlier after the treatment of MCF-7 cells with (campthothecin-20(s)-O-(2-pyrazul-l) acetic ester (CPT6) [34] and HepG2 cells with PAMAM/Flag-apoptin [35]. Changes in chromatin condensation and fragmentation might be an early sign of apoptosis [36,37] or a direct interaction of cationic hybrid dendrimers with negatively charged DNA [29,38]. Lazniewska et al. [16,30] reported that mHippoE-18 [30] and N2a [16] cells exposed to VPD (viologen–phosphorus dendrimers) maintain normal morphology, whereas CPD dendrimers (cationic–phosphorus dendrimer) disturbed the normal morphology in mHippoE18 and N2a cell lines [29].

Since none of these methods explicitly define the mechanism of cell death, further tests were performed. To assess the fraction of apoptotic and necrotic cells, two methods based on double staining were used. Confocal analysis of OA/EB stained and flow cytometry analysis of Annexin-V/PI stained samples confirmed a higher toxicity of SMT2 than SMT1. However, the data show that SMT2 most likely activates the apoptotic pathway. The above-described alterations in N2a cells after their treatment with 5 μM SMT1 are in agreement with the results from the double staining methods. The data confirm a slightly higher toxicity of SMT1 to N2a cells than mHippoE-18 cells. Nevertheless, the mechanism of action of hybrid dendrimers is probably through inducing apoptosis.

Contrary to VPD, hybrid dendrimers have a positive charge both on the surface and the inside [39]. SMT1 and SMT2 hybrid dendrimers have a rigid inner structure containing 12 positive charges, and their surface charges are +12 and +24, respectively [9,18]. Thus, they can interact with the plasma membrane, which explains the presence of lamellar bodies, numerous secondary lysosomes and multi-vesicular bodies in the cells treated with these dendrimers, as well as changes in the mitochondria ultrastructure.

Dendrimers are immediately coated with proteins in biological fluids, forming a so-called “protein corona”, and can enter a cell via clathrin-dependent endocytosis and/or macropinocytosis [16,35,39,40,41,42,43,44,45,46]. Solarska-Sciuk [43] demonstrated the presence of diamond nanoparticles in endocytic vesicles in Huvec-ST and A549 cell lines.

We also postulated that positively charged dendrimers acting on negatively charged plasma membranes could contribute to the formation of nanoholes in the membrane, which can lead to cellular dysfunction or death [16]. The ultrastructure of N2a cells in the presence of the SMT2 dendrimer was significantly altered. This indicates the loss of membrane integrity, most likely resulting in decreased cell viability.

## 5. Conclusions

The surface flexibility for hybrid dendrimers increases with dendrimer generation [18]. Our results show that both dendrimers at low concentrations affected a cancer cell line more than a normal one. Also, generation-dependent effects were found: the highest generation of dendrimer (SMT2) induced a greater cytotoxic effect and morphological modifications. The SMT1 dendrimer can reach mitochondria and did not induce significant cytotoxic effects for normal or cancerous cells. Greater toxicity of the SMT2 dendrimer was probably related to its outer positive charges, with a significant effect caused by the internal ones [9]. Thus, SMT1 seems to have potential as a nanocarrier for mitochondria, while SMT2 may have potential as an anti-cancer drug per se in CNS disorders. However, the safety of SMT1 still needs more investigation before it can be accepted as a low-toxic compound for non-cancerous cells.

## Figures and Tables

**Table 1:** Apoptosis study on mHippoE-18 and N2a cells after 24 h exposure to SMT 1 and 2 dendrimers at 5μM. Analysis was performed using orange acridine/ethidium bromide (OA/EB) double staining. Performed data were collected from at least 250 counted cells per sample (*n* = 3; * *p* < 0.05; ↑/↓ = a statistically significant increase/decrease compared to the control; # = a statistically significant difference between N2a and mHippoE-18).

	mHippoE-18			N2a		
	Control	SMT1	SMT2	Control	SMT1	SMT2
Healthy	98.49 ± 0.85	95.89 ± 2.69	49.81 ± 6.34 *↓	98.37 ± 1.2	83.01 ± 7.87 *#↓	28.57 ± 1.59 *#↓
Necrotic	0.39 ± 0.88	0.54 ± 1.20	2.56 ± 7.69	0.20 ± 0.44	0.33 ± 0.8	1.56 ± 3.13
Early apoptotic	0.39 ± 0.87	2.55 ± 2.36	45.41 ± 13.04 *↑	0.39 ± 0.88	15.95 ± 7.7 *#↑	68.68 ± 13.39 *↑
Late apoptotic	0.72 ± 0.99	1.03 ± 1.41	2.22 ± 6.67	1.04 ± 1.49	0.67 ± 1.03	1.19 ± 1.34

**Table 2:** Apoptosis study on mHippoE-18 and N2a cells after 24 h exposure to 5 µM SMT 1 and 2 dendrimers. Flow cytometry analysis was performed using annexin V and propidium iodide stains (*n* = 3; * *p* < 0.05; ↑/↓ = a statistically significant increase/decrease compared to control; # = a statistically significant difference between N2a and mHippoE-18).

		mHippoE-18			N2a	
	Control	SMT1	SMT2	Control	SMT1	SMT2
Healthy	94.06 ± 1.00	77.16 ± 5.76 *↓	18.92 ± 3.21 *↑	97.67 ± 1.03	61.63 ± 5.13 *#↓	16.08 ± 4.18 *↓
Necrotic	3.97 ± 0.76	12.48 ± 5.08 *↑	16.52 ± 3.98 *↑	1.42 ± 0.52	15.07 ± 1.51 *↑	14.61 ± 1.38 *↑
Early apoptotic	1.06 ± 0.93	1.54 ± 0.45	0.73 ± 0.48	0.44 ± 0.15	2.55 ± 0.39	1.74 ± 0.52
Late apoptotic	0.91 ± 0.42	8.82 ± 1.62 *↑	63.84 ± 7.62 *↑	0.48 ± 0.29	20.75 ± 5.37 *#↑	67.58 ± 5.46 *↑
